# Interpopulation differences and temporal synchrony in rates of adult survival between two seabird colonies that differ in population size and distance to foraging grounds

**DOI:** 10.1002/ece3.10455

**Published:** 2023-10-03

**Authors:** C. Horswill, V. Warwick‐Evans, N. P. G. Esmonde, N. Reid, H. Kirk, K. R. Siddiqi‐Davies, S. A. Josey, M. J. Wood

**Affiliations:** ^1^ ZSL Institute of Zoology London UK; ^2^ Department of Genetics, Evolution and Environment, Centre for Biodiversity and Environmental Research University College London London UK; ^3^ British Antarctic Survey Cambridge UK; ^4^ School of Biological Sciences Queen's University Belfast Belfast UK; ^5^ Interdisciplinary Conservation Science Group, Centre for Urban Research RMIT University Melbourne Victoria Australia; ^6^ Department of Zoology University of Oxford Oxford UK; ^7^ National Oceanography Centre Southampton UK; ^8^ University of Gloucestershire Cheltenham UK

**Keywords:** carry‐over effects, demography, foraging ecology, mark‐recapture, synchrony

## Abstract

Understanding the processes that drive interpopulation differences in demography and population dynamics is central to metapopulation ecology. In colonial species, populations are limited by local resource availability. However, individuals from larger colonies will travel greater distances to overcome density‐dependent competition. Consequently, these individuals may also experience greater carry‐over effects and interpopulation differences in demography. To test this prediction, we use mark‐recapture data collected over four decades from two breeding colonies of a seabird, the Manx shearwater (*Puffinus puffinus*), that exhibit strong spatial overlap throughout the annual cycle but differ in population size and maximum foraging distances. We quantify interpopulation differences and synchrony in rates of survival and assess whether local mean wind speeds act to strengthen or disrupt synchrony. In addition, we examine whether the imputed interpopulation differences in survival can generate population‐level consequences. The colony where individuals travel further during the breeding season had slightly lower and more variable rates of survival, indicative of individuals experiencing greater carry‐over effects. Fluctuations in survival were highly synchronous between the colonies, but neither synchronous, nor asynchronous, variation could be strongly attributed to fluctuations in local mean wind speeds. Finally, we demonstrate that the imputed interpopulation differences in rates of survival could lead to considerable differences in population growth. We hypothesise that the observed interpopulation differences in rates of adult survival reflect carry‐over effects associated with foraging distances during the breeding season. More broadly, our results highlight that breeding season processes can be important for understanding interpopulation differences in the demographic rates and population dynamics of long‐lived species, such as seabirds.

## Graphical abstract

Populations that spatially overlap on foraging grounds can experience divergent costs if factors determining access to resources differ. To test this prediction, we used mark‐recapture data to quantify interpopulation differences and synchrony in rates of survival between two colonies of a trans‐Atlantic migratory seabird that exhibit strong spatial overlap throughout the annual cycle but differ in population size and distance to foraging grounds.

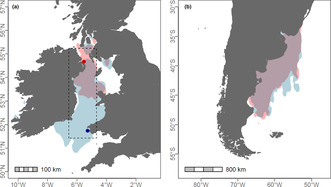

## 1 INTRODUCTION

Understanding the mechanisms that generate interpopulation differences in demography and population dynamics is central to metapopulation ecology. Populations of the same species may vary in their mean demographic rates, as well as exhibit differing levels of temporal variation in these processes (Frederiksen et al., 2005; Horswill et al., 2019). Additionally, distinct populations may fluctuate in size and demography with varying degrees of synchrony over different spatial scales (Lindstrom et al., 1996; Sinclair, 1993; Steen et al., 1996; Sutcliffe et al., 1996). Such variation is often attributed to spatial autocorrelation in the relationships linking demographic rates with environmental variables (Grosbois et al., 2009; Sæther et al., 2006; Stenseth et al., 1999), or large‐scale weather patterns generating regionally correlated population fluctuations (Hanski & Woiwod, 1993; Lindstrom et al., 1996; Sinclair, 1993). However, studies reporting these relationships rarely investigate the possible mechanisms connecting environmental covariates to interpopulation differences in demography.

Ashmole's halo hypothesis predicts that populations of colonial species are limited by local resource availability (Ashmole, 1963). In this framework, density‐dependent competition increases with population size forcing individuals to travel further to balance their self‐feeding and offspring provisioning requirements. This mechanistic link between population size, food availability and foraging behaviour has been repeatedly proposed in colonial seabirds (e.g. Ballance et al., 2009; Jovani et al., 2016; Lewis et al., 2001; Wakefield et al., 2013). However, seabirds will also increase their average trip duration and distance during years with adverse prey or climatic conditions (Burke & Montevecchi, 2009; Campbell et al., 2019; Horswill et al., 2017). In this context, individuals from large colonies, which are already travelling further and potentially working harder to balance energy allocation to parent and offspring survival, are likely to experience greater energetic and reproductive costs, compared to their counterparts from smaller colonies (e.g. Fayet et al., 2021).

Energetic costs occurring in one season can influence how well individuals perform (i.e., reproduce or survive) in subsequent seasons (Fayet et al., 2016; Harrison et al., 2011; Inger et al., 2010). For example, costs incurred during reproduction can effect changes in an organism's state, such as its energy reserves (e.g. Dawson et al., 2000), thereby, influencing subsequent survival (e.g. Daan et al., 1996; Wanless et al., 2023). Such downstream consequences of resource allocation, often referred to as “carry‐over effects”, can thereby generate major demographic consequences. Moreover, interpopulation variation in these carry‐over effects can lead to interpopulation differences in demography (Wilson et al., 2022), and, potentially, population dynamics.

**FIGURE 1:**
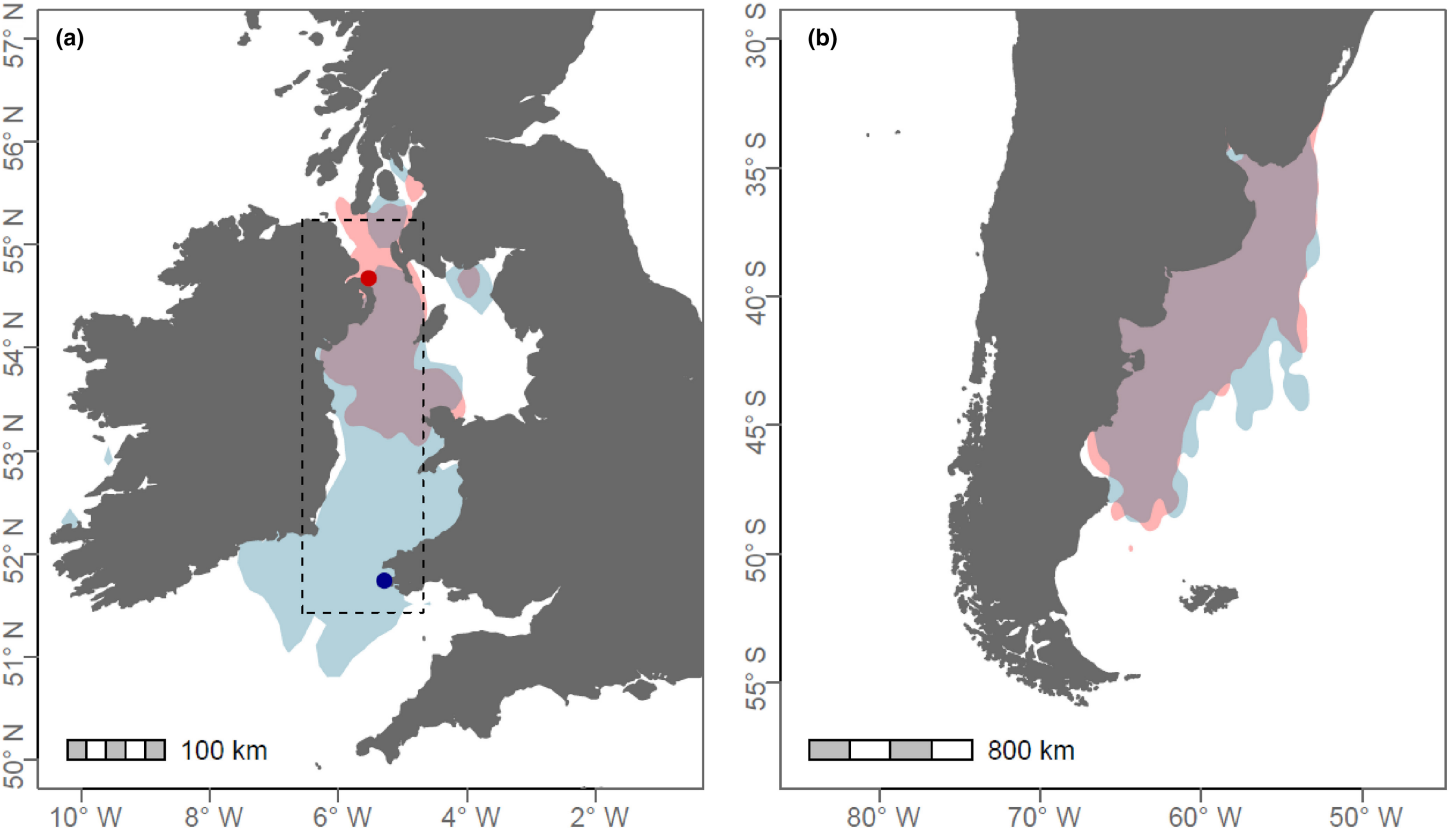
Manx shearwaters breeding on Copeland (red) and Skomer Island (blue) demonstrate a strong spatial overlap in foraging habitat use during the (a) breeding season and (b) overwintering period. Dashed grid square in panel (a) shows the area used to extract summer wind speed data. Figure adapted from Dean et al. (2015) and Kirk (2017). Kernel densities were calculated using 90% occupancy and (a) 35 birds for Copeland and 69 birds for Skomer Island, and (b) 13 birds for Copeland and 26 birds for Skomer Island.

In this study, we quantify interpopulation differences and temporal synchrony in rates of adult survival between two colonies of a long‐lived seabird, the Manx shearwater (*Puffinus puffinus*). While the study colonies exhibit strong temporal and spatial overlap throughout the annual cycle (Figure 1; also see Dean et al., 2015; Kirk, 2017), they diverge in terms of population size and the maximum foraging distances that individuals travel during the breeding season. Despite both colonies targeting similar foraging grounds, the smaller colony exhibits considerably shorter maximum foraging distances (Figure 1a; Dean et al., 2015). We also examine how climate contributes to interpopulation synchrony in rate of survival using a variable previously identified as influential to Manx shearwaters, summer mean wind speed (Wood et al., 2021). Similar to many other bird species, Manx shearwaters employ a flight strategy characterised by bursts of flapping flight interspersed with gliding phases (Tobalske, 2001). Flapping duty cycles (representing the fraction of time spent actively beating the wings) increase during strong headwinds until near‐continuous flapping is exhibited (Spivey et al., 2014). Consequently, we hypothesise that individuals from the larger colony, where maximum foraging distances are longer on average, will experience more pronounced carry‐over effects and lower rates of adult survival, particularly during years with elevated mean wind speeds. In this context, we anticipate that elevated wind speeds will contribute to desynchronising rates of adult survival between the two colonies.

## 2 METHODS

### 2.1 Study species

The Manx shearwater is a pelagic burrow‐nesting seabird that reproduces once per year during the boreal summer. Most of the global population breed in the UK and Ireland, restricted to a small number of colonies on the Atlantic coast (BirdLife International, 2022). Individuals forage under central place constraint during chick rearing, travelling up to ca. 85 km from the colony each day (Dean et al., 2015). Birds then undertake trans‐equatorial, trans‐Atlantic migration, converging on a restricted area that is close to the Argentinean coast, south of the Rio de la Plata (Figure 1b; Guilford et al., 2009; Kirk, 2017). Adults are highly site faithful once they start breeding, and any movements within a colony are usually short (Harris, 1966; Perrins et al., 1973). Natal dispersal between colonies also appears to be low (Harris, 1972).

### 2.2 Demographic data

Our analysis included mark‐recapture data collected from two colonies of Manx shearwater breeding in the UK: (1) Copeland Islands, Northern Ireland (54.43° N, 3.39° W) and (2) Skomer Island, Wales (51.74° N, 5.30° W; Figure 1). These colonies combined represent approximately 41% of the global breeding population. Copeland is estimated to support 4850 breeding pairs of Manx shearwaters (Stewart & Leonard, 2007), and Skomer Island is estimated to support 350,000 breeding pairs (Perrins et al., 2018). In line with predictions based on Ashmole's halo hypothesis (Ashmole, 1963), maximum foraging distances during the breeding season are also considerably shorter for individuals from Copeland, compared to their counterparts from Skomer Island: 72% shorter during incubation and 42% shorter during chick‐rearing (Figure 1a; Dean et al., 2015). Mark‐recapture monitoring of Manx shearwaters began in 1952 at Copeland and in 1977 on Skomer Island. Therefore, to examine interpopulation synchrony in rates of apparent adult survival, we restricted these datasets to the 44‐year period with concurrent data: 1977–2020.

In both study locations, breeding Manx shearwaters were ringed under licence using hard‐metal British Trust for Ornithology (BTO) rings. Ringing and resighting data were predominantly collected during the beginning of the breeding season. Adult birds were selected for ringing by capturing birds from breeding burrows and from the ground at night. This methodology introduces many potentially transient or prospecting individuals into the dataset. Therefore, to minimise individual heterogeneity in rates of survival, we removed the large number of individuals not observed again in the years following first release (Copeland: *n* = 7009; 55% of individuals, and Skomer Island: *n* = 440; 27% of individuals). These individuals may reflect a large pool of non‐breeding adults, or, alternatively, that birds recruit outside of the immediate study plots. Recorded movements of ringed individuals between the two study colonies are very limited (CBO, 2001; M. Wood personal observations, C. Acheson personal communications) and therefore assumed not to influence survival estimation (e.g., Horswill, Wood, et al., 2022). The final dataset included 4849 individuals ringed on Copeland and 1164 individuals ringed on Skomer Island. The sex of individual birds is unknown and therefore not included in our analyses. Individuals marked on Copeland before 1977 and resighted after 1977 (*n* = 590) were included by reassigning the year of first release in these instances as 1977. Any heterogeneity in recapture rates introduced by this cohort were isolated by modelling the recapture process as fully time varying with trap‐dependence.

### 2.3 Synchrony in rates of adult survival

We used a state‐space formulation of the Cormack‐Jolly‐Seber (CJS) model (Gimenez et al., 2007) to estimate rates of apparent adult survival from the mark‐recapture data. The resighting interval was 1 year, from the end of the breeding season in year *t–*1 to the end of the breeding season in year *t*. In the state‐space CJS model, annual survival events were modelled as the state process and annual recapture probabilities were modelled as the observation process. Both processes were imputed using Bernoulli distributions with a logit link function. Initial goodness‐of‐fit testing conducted in program MARK (v.9.x, White & Burnham, 1999) using the RELEASE program (Burnham et al., 1987) identified trap‐dependence (i.e., capture heterogeneity) in the data. Trap‐dependence in mark‐recapture data can lead to an underestimation of survival (Sandland & Kirkwood, 1981), and to mitigate this, we incorporated a trap response in the CJS model (i.e., trap‐dependent recapture rates). The functions for annual apparent survival (ϕ) and recapture (*p*) for each colony (*c*) and year (*t*) were:

(1) $$\begin{array}{l} \text{logit}\left(\phi_{c,t}\right)=\alpha_{c}+\gamma_{t}+\varepsilon_{\phi ,c,t}\\ \text{logit}\left(p_{c,t}\right)=\delta_{c,k}+\varepsilon_{p,c,t} \end{array}$$

Here, *α* and *δ* are the intercept terms for adult survival and recapture probabilities, respectively, whereby the recapture term is modelled as a function of whether or not an individual was captured in the preceding year (*δ*_*c*,1_ = Yes, *δ*_*c*,2_ = No; Kéry & Schaub, 2012). All intercept terms were transformed to the logit scale from uniform prior distributions bound between zero and one. To capture interpopulation synchrony and asynchrony in survival rates we used random effect terms based on normal distributions. These were common across the two colonies for the synchronous term: $\gamma_{t}\sim N\left(0,\sigma_{\gamma }^{2}\right)$, and colony‐specific for the asynchronous term: $\varepsilon_{\phi ,c,t}\sim N\left(0,\sigma_{\phi ,\varepsilon }^{2}\right)$. This colony‐specific structure was also replicated to assign annual fluctuations in recapture rates: $\varepsilon_{p,c,t}\sim N\left(0,\sigma_{p,\varepsilon }^{2}\right)$. We assigned the standard deviations for the three random effects on the logit scale using uniform prior distributions bound between 0.1 and 10 (see Figure S1 for an illustration of the observed temporal variation resulting from the minimum value, i.e., 0.1).

To quantify synchronous variation in rates of adult survival, we calculated the intra‐class correlation (ICC, Equation 2). Here, the ICC reflects the proportion of total variance explained by the synchronous term in the survival function (Equation 1) (e.g. Grosbois et al., 2009; Lahoz‐Monfort et al., 2011; Reiertsen et al., 2021; Schaub et al., 2015):

(2) $$\mathrm{ICC}=\frac{\sigma_{\gamma }^{2}}{\sigma_{\gamma }^{2}+\sigma_{\phi ,\varepsilon }^{2}}$$

An ICC score close to zero indicates that the local scale asynchronous variance term is large relative to the global scale synchronous term: i.e., that between‐year variance is largely asynchronous between the colonies. By contrast, an ICC score close to one indicates that the opposite is true, and between‐year variance is largely synchronous between the colonies.

**FIGURE 2:**
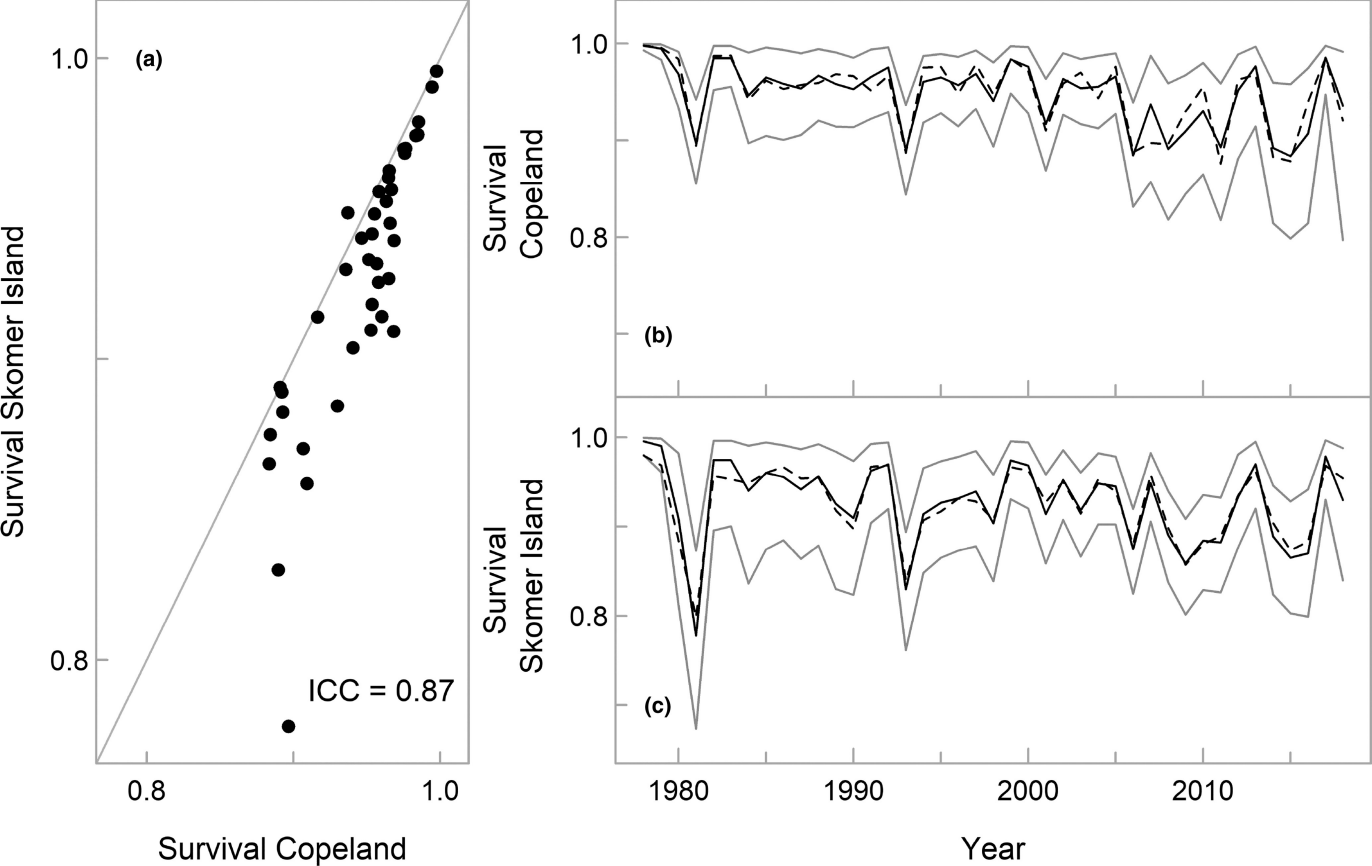
(a) Manx shearwaters from Copeland and Skomer Island demonstrated highly synchronous rates of apparent adult survival (ICC = 0.87, 95% CRI: 0.53–0.98). Grey diagonal line shows the 1:1 correlation between colonies for reference. Median posterior values of survival shown. Survival was slightly higher and less variable at (b) Copeland, compared to (c) Skomer Island. Grey lines in panels (b) and (c) represent the 95% credible intervals. In all panels, the survival estimates shown were obtained from the CJS model including summer wind speed as a covariate. In panels (b) and (c), survival probabilities estimated using separate colony‐specific time‐dependent models are also shown as dashed lines for examination of model fit.

We fitted the CJS model using a Bayesian approach in JAGS (v. 4.3.0) (Plummer, 2003) via the “jagsUI” library (v 1.5.1) (Kellner, 2019) for program R (v. 4.0.2, R Core Team, 2020). We specified the prior distributions using biologically driven bounds (see Table S1 for details on all prior distributions). Model fitting involved running three Monte Carlo Markov chains (MCMC) for 1 × 10^5^ iterations and retaining every 100th step to minimise autocorrelation in the MCMC sampling. To confirm convergence of the chains we used the Brooks‐Gelman‐Rubin diagnostic tool (all values $\hat{r}$ ≤ 1.01) and the effective sample size of the MCMC chains for each parameter (all values ≥ 300). Trace plots of the MCMC chains for imputed vital rates and parameters are provided in the supplementary information to show convergence (Figures S2–S8). We removed the first 5000 MCMC draws as burn‐in, and visually checked that convergence of the MCMC chains had occurred before this cut off. We constructed additional independent CJS models for each colony (i.e., identical structure to Equation 1 but without *γ*_*t*_) to demonstrate that including the synchronous random effect term did not influence the imputed rates of apparent survival (Appendix A1 in Appendix S1, Figure 2). Finally, we report the median posterior values of parameters imputed by the state‐space CJS model, and detail annual values of survival in Table S2.

### 2.4 Contribution of wind to synchrony and asynchrony in rates of survival

To examine how climate contributed to synchrony in rates of adult survival, we used a variable previously identified as influential to the survival rates of adult Manx shearwaters at Skomer Island, i.e., summer mean wind speeds (Wood et al., 2021; Figure 1a). Here, the proposed mechanism is that conditions during the summer, breeding season indirectly influence survival during the subsequent winter through sustained carry‐over effects. Summer mean wind speeds (m/s) were calculated as the hypotenuse of mean northerly and mean easterly wind speeds, calculated from monthly averages available from NOAA's NCEP‐NCAR CDAS‐1 Reanalysis data set (Kalnay et al., 1996). Data are available at http://iridl.ldeo.columbia.edu/SOURCES/.NOAA/.NCEP‐NCAR/.CDAS‐1/.MONTHLY. These data are produced on a 2.5 × 2.5 degree grid and we extracted values for the two grid squares that combine the foraging area utilised by Manx shearwaters from Copeland and Skomer Island during the summer months, i.e., May to August (Figure 1a; Dean et al., 2015). Summer mean wind speeds were positively correlated between the two grid squares (Spearman correlation coefficient = 0.86), although were often slightly stronger in the square closest to Skomer Island (Figure S9). To ensure identifiability between the intercept and slope coefficient terms in the CJS model, we averaged the wind speed data over the two squares and centred it on zero by subtracting the mean (Ogle & Barber, 2020).

We quantified the contribution of summer wind speeds to synchrony in Manx shearwater survival in two steps. Firstly, we adapted the survival function in the CJS model (Equation 1) to incorporate the summer wind speed covariate (*W*_*t*_):

(3) $$\text{logit}\left(\phi_{c,t}\right)=\alpha_{c}+\beta_{c}W_{t}+\gamma_{t}+\varepsilon_{\phi ,c,t}$$

Here, each colony‐specific coefficient describing the linear relationship between adult survival and summer wind speed (*β*_*c*_) was assigned from a normal prior distribution centred on zero with a standard deviation of 1. Previous analysis on Manx shearwaters indicates that the relationship between rates of adult survival and summer wind speed is negative, whereby higher winds are associated with lower rates of survival (Wood et al., 2021). However, the time series considered in our study spans a considerably longer time period (1977–2020 vs. 1993–2019) and a larger range of summer wind speeds (1.14–3.37 m/s vs. 1.80–3.37 m/s). Therefore, we allowed the direction and strength of the colony‐specific relationships with wind to be determined during model fitting, i.e., centring the prior distributions for the wind speed coefficients on zero. Next, to measure the influence of summer wind speed in generating synchrony and asynchrony in rates of survival, we compared the residual variance imputed by the random effects in the CJS models with [$\sigma_{\gamma }^{2}\left(\mathrm{res}\right)$, $\sigma_{\phi ,\varepsilon }^{2}\left(\mathrm{res}\right)$] and without [$\sigma_{\gamma }^{2}\left(\text{total}\right)$, $\sigma_{\phi ,\varepsilon }^{2}\left(\text{total}\right)$] wind speed included (e.g. Grosbois et al., 2009; Lahoz‐Monfort et al., 2011). Here, we estimated how summer wind speed in the breeding foraging area influences the intercolony synchronous term of between year variance as:

(4) $$\omega_{\gamma }=1-\frac{\sigma_{\gamma }^{2}\left(\mathrm{res}\right)}{\sigma_{\gamma }^{2}\left(\text{total}\right)}$$

and the asynchronous term of between year variance as:

(5) $$\omega_{\varepsilon }=1-\frac{\sigma_{\phi ,\varepsilon }^{2}\left(\mathrm{res}\right)}{\sigma_{\phi ,\varepsilon }^{2}\left(\text{total}\right)}$$

Examination of the relationship between imputed rates of survival and summer wind speeds indicated a potential non‐linear relationship. We tested this post‐hoc due to the large amount of residual variation in the CJS model including wind speed. Using the statistical package “segmented” (v 1.6.2, Muggeo, 2008) in program R (v. 4.1.2, R Core Team, 2020), we fitted a piece‐wise linear model with one breakpoint to the posterior median values of survival. Here, sensitivity of the estimated breakpoint to starting values was evaluated using bootstrap restarting (Wood, 2001) and we retained the default number of bootstrap samples (*n* = 10; Muggeo, 2008). To estimate the confidence interval of the breakpoint, we used the Delta method.

### 2.5 Population projection modelling

We constructed Leslie matrix models for each colony to test whether the imputed interpopulation differences in survival can generate population‐level consequences. Each Leslie matrix model was parameterised using a full colony‐specific time series of survival rates. We then projected the population dynamics for each colony over the duration of the survival data. To standardise the Leslie matrix models, we assigned an identical constant rate of fecundity (*f* = 0.64, Wood et al., 2021) and breeding propensity (*b* = 0.87, Wood et al., 2021) to both colonies. These values reflect the demographic profile of Manx shearwaters breeding on Skomer Island that exhibit elevated rates of fecundity and breeding propensity (Wood et al., 2021). Like many seabirds, Manx shearwaters are largely unobservable during the first years of life and estimates of juvenile survival (i.e., during the first year post fledging $\phi_{j,t}$) are lacking (Horswill & Robinson, 2015). Juvenile survival of seabirds is typically lower than that of adults (Horswill & Robinson, 2015) and additive across age classes (Cam et al., 2005; Horswill et al., 2014). Therefore, to estimate juvenile survival for each year and colony, we applied a theoretical 50% scalar to the imputed time series of adult survival.

The Leslie matrix models followed a pre‐breeding census, predicting colony size (*N*) in year *t* + 1 as a function of colony size in year *t* (Equation 6). Following published estimates, we assumed that individuals start breeding at age 5 years (Perrins et al., 1973). We also assumed a 1:1 sex ratio in the colony and halved fecundity to model female numbers only. In all models, female birds survived from egg laying to chick fledging with probability $\frac{f}{2}$ and from fledging to age 1 year with probability $\phi_{j,t}$. Therefore, the juvenile age class (0–1 years) was assigned the following combined survival probability: $\frac{f}{2}\phi_{j,t}$. Birds then survived the immature age classes (1–2 years) with a probability equivalent to that of breeding adults ($\phi_{a,t}$). Once in the breeding age class, individuals reproduced annually with a breeding propensity of *b*.

(6) $${\left[\begin{array}{c} N_{1}\\ N_{2}\\ N_{3}\\ N_{4}\\ N_{5} \end{array}\right]}_{t+1}=\left[\begin{array}{ccccc} 0 & 0 & 0 & 0 & b\frac{f}{2}\phi_{j,t}\\ \phi_{a,t} & 0 & 0 & 0 & 0\\ 0 & \phi_{a,t} & 0 & 0 & 0\\ 0 & 0 & \phi_{a,t} & 0 & 0\\ 0 & 0 & 0 & \phi_{a,t} & \phi_{a,t} \end{array}\right]{\left[\begin{array}{c} N_{1}\\ N_{2}\\ N_{3}\\ N_{4}\\ N_{5} \end{array}\right]}_{t}$$

To account for parameter uncertainty in survival estimates, we ran each Leslie matrix model for 1000 iterations and randomly assigned complete time series of survival to each iteration from the joint posterior distribution of the CJS model. We initiated each model run with 4000 breeding pairs and estimated the initial stable age distribution for each colony using the dominant eigenvector of the Leslie matrix based on mean vital rates (Caswell, 2001). We also incorporated demographic stochasticity using binomial distributions on survival events.

To compare the resulting population trajectories, we used the finite rate of annual population growth, estimated using the dominant eigenvalue of the annual Leslie matrices (Caswell, 2001). We also used the annual counterfactual of population size, calculated as the ratio of annual population sizes (Jitlal et al., 2017). We constructed and ran all population modelling in program R (v. 4.0.2, R Core Team, 2020).

## 3 RESULTS

### 3.1 Synchrony in rates of adult survival

The median posterior values of the intercept terms in the survival function (i.e., geometric means) of the CJS model, indicated that survival was slightly higher at Copeland, compared to Skomer Island, albeit with overlapping 95% credible intervals (Table 1). We also found that a high proportion of annual variation in survival was synchronous between the colonies (ICC = 0.87, 95% CRI: 0.53–0.98; Figure 2a), and that total temporal variation was slightly lower on Copeland (SD = 0.03), compared to Skomer Island (SD = 0.04; Figure 2b,c). At both colonies, the mean recapture probability was higher if an individual was seen the year before (Table 1), however, recapture probabilities were consistently lower on Copeland than on Skomer Island (Table 1).

**TABLE 1:** Posterior median values (with 95% credible intervals) for the parameters of the CJS model including summer wind speeds.

Parameter	Copeland	Skomer Island
*α*	0.96 (0.94 to 0.97)	0.94 (0.91 to 0.96)
*δ*_1_	0.25 (0.21 to 0.28)	0.69 (0.65 to 0.73)
*δ*_2_	0.16 (0.14 to 0.19)	0.23 (0.19 to 0.27)
*β*	−0.19 (−0.61 to 0.22)	−0.23 (−0.61 to 0.16)

*Note*: Intercept values (geometric mean values) of survival (*α*) and recapture probabilities for individuals recaptured (*δ*_1_) and not recaptured (*δ*_2_) the year before, as well as the coefficient of the linear term with summer wind speeds (*β*).

### 3.2 Contribution of wind to synchrony and asynchrony in rates of survival

**FIGURE 3:**
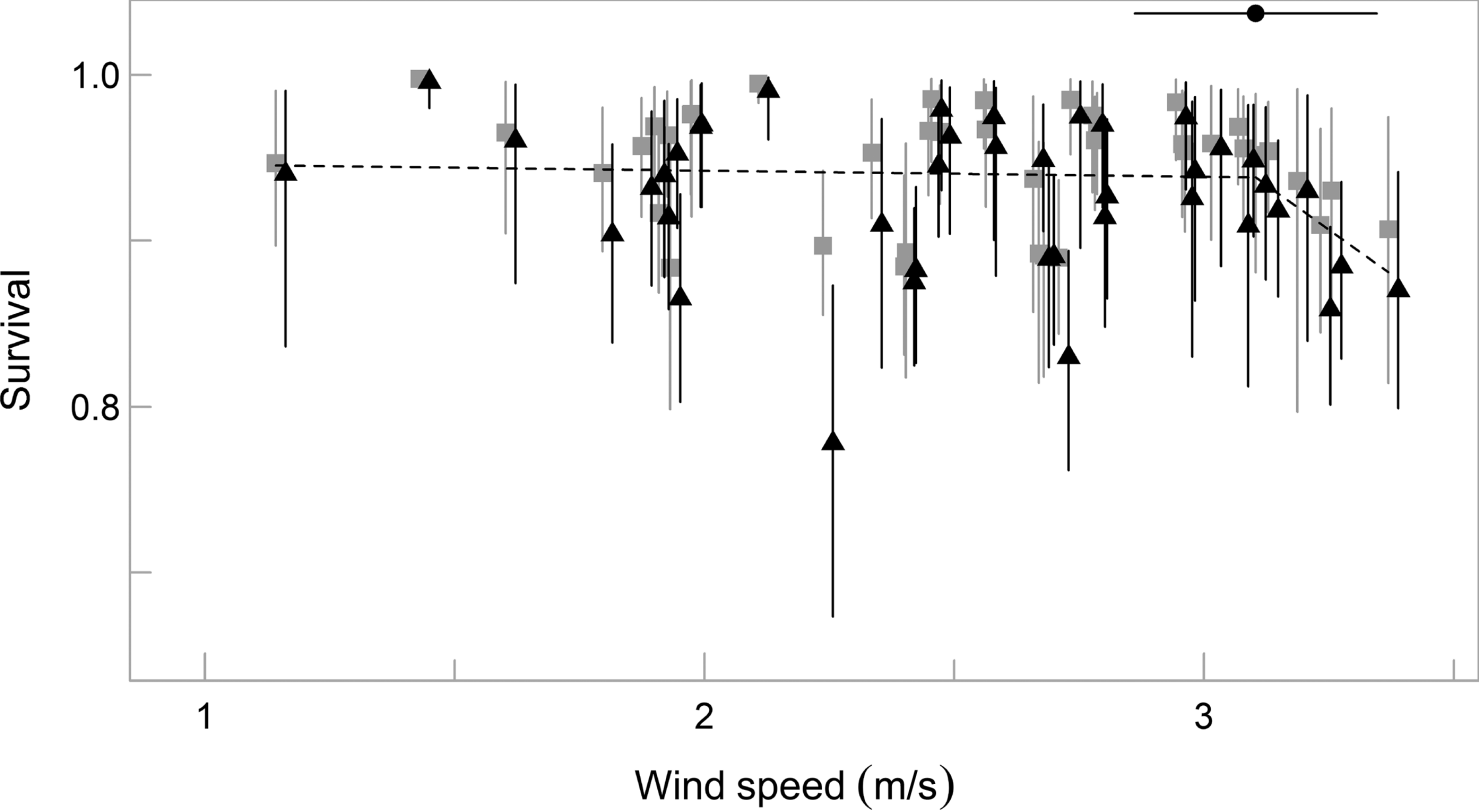
Summer wind speeds did not have a strong influence on Manx shearwater rates of adult survival at Copeland (grey square) or Skomer Island (black triangle) (Table 1). However, a piece‐wise regression analysis (dashed black line) indicates that a negative effect of wind may occur above wind speeds of 3.04 m/s (95% CI: 2.86, 3.35; breakpoint estimate with horizontal 95% CI shown at top of graph). The survival estimates were obtained from the CJS model including summer wind speed as a covariate, vertical error bars represent the 95% credible intervals.

Based on the posterior credible intervals of the slope coefficients, summer wind speeds demonstrated a similar effect on Manx shearwater survival at both colonies (Table 1, Figure 3). In both cases, the 95% credible intervals included zero, however, the full posterior distributions were highly negatively skewed with 82.6% and 88.1% of the MCMC draws below zero for Copeland and Skomer Island, respectively (Figure S10).

Comparing the median posterior values of the synchronous variance terms for CJS models with and without wind included, indicated that summer wind speed accounted for 3% of synchronous variation in survival (Table 2). However, given the highly similar 95% credible intervals of the synchronous variance terms, we conclude that the imputed difference in the median values reflects model stochasticity. Likewise, the 95% credible intervals of the two asynchronous variance terms were highly similar, such that the summed median global and local random variance for models with and without wind speed were identical (1.18, Table 2). Consequently, it appears that neither synchronous, nor asynchronous, variation in Manx shearwater survival can be strongly attributed to fluctuations in summer wind speed (Table 2).

**TABLE 2:** Imputed residual variance of the random effects from the CJS models excluding (total) and including (residual) wind speed (*W*).

Model	Variances	Synchronous variance ($\sigma_{\gamma }^{2}$)	Asynchronous variance ($\sigma_{\varepsilon }^{2}$)	Interclass correlation (ICC)
$\phi \left(\alpha_{c}+\gamma_{t}+\varepsilon_{\phi ,c,t}\right)p\left(\delta_{c,k}+\varepsilon_{p,c,t}\right)$	Total	1.05 (0.47, 2.18)	0.13 (0.03, 0.49)	0.89 (0.55, 0.98)
$\phi \left(\alpha_{c}+\beta_{c}W_{t}+\gamma_{t}+\varepsilon_{\phi ,c,t}\right)p\left(\delta_{c,k}+\varepsilon_{p,c,t}\right)$	Residual	1.02 (0.44, 2.14)	0.16 (0.03, 0.54)	0.87 (0.53, 0.98)

*Note*: The amount of synchronous variation in rates of adult survival for Manx shearwaters breeding on Copeland and Skomer Island (i.e., the intra‐class correlation, ICC) is also shown.

The piece‐wise regression analysis indicated a potential non‐linear relationship between summer wind speed and survival, whereby survival decreases (slope coefficient = −0.22, SE = 0.16, *t* = −1.35) at wind speeds above 3.04 m/s (95% CI: 2.86, 3.35; Figure 3).

### 3.3 Population projection modelling

**FIGURE 4:**
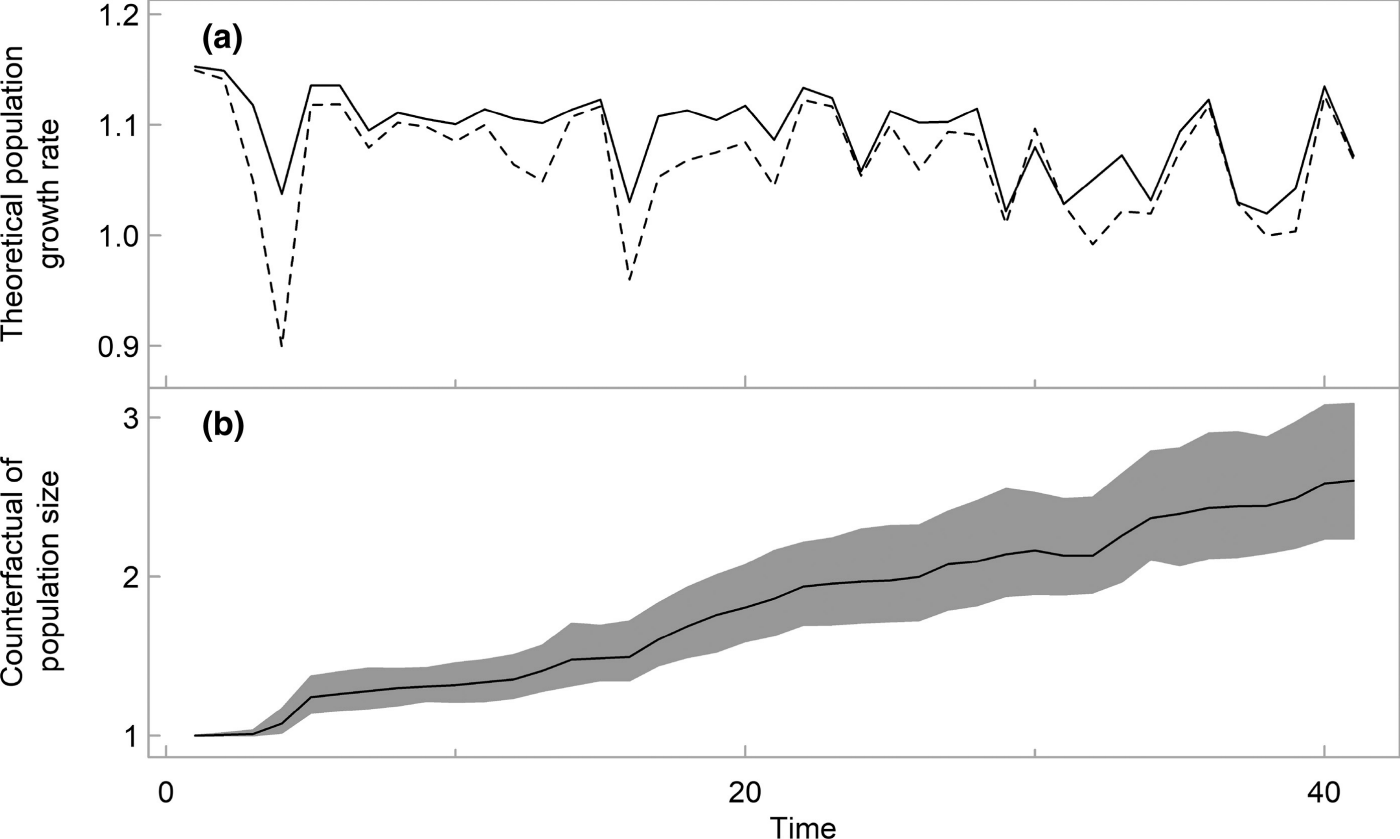
(a) Theoretical annual population growth rates based on the colony‐specific time series of survival were predominantly faster for Copeland (solid line), compared to Skomer Island (dashed line). (b) This resulted in a final mean population size on Copeland that was approximately 2.6 times larger than on Skomer Island. Annual counterfactuals of population size based on the colony‐specific mean projected population sizes (black line) shown with the annual counterfactuals of population size based on the 95% quantile in projected population sizes (grey polygon).

We ran colony‐specific Leslie matrix models to quantify the theoretical population response to the imputed interpopulation differences in rates of survival. This analysis demonstrated that despite a seemingly small difference in the colony‐specific mean values (Table 1), the time series of survival rates imputed for Copeland generates predominantly faster annual rates of population growth, compared to Skomer Island (Figure 4a). The projected absolute population size for Copeland was approximately 2.60 times (95% CI: 2.24, 3.09) larger than Skomer Island after 40 years (Figure 4b).

## 4 DISCUSSION

In this study, we examined interpopulation differences and synchrony in rates of adult survival for two colonies of a pelagic seabird, the Manx shearwater. These colonies exhibit strong spatial overlap throughout the annual cycle, yet they diverge in terms of population size and maximum foraging distances (Figure 1; Dean et al., 2015; Kirk, 2017). In agreement with results from previous studies investigating synchrony in rates of survival for migratory birds that spatially overlap (e.g. Reiertsen et al., 2021; Reynolds et al., 2011; Schaub et al., 2005), we found a high degree of synchrony between the two colonies. However, we also show that Manx shearwaters breeding on Skomer Island, which travel further during the breeding season (Dean et al., 2015), have slightly lower and more variable rates of adult survival. We also found evidence that Manx shearwater rates of survival are negatively influenced during years with elevated summer wind speeds. However, we did not identify that wind speed acts to strengthen or disrupt temporal synchrony in rates of survival, although this may reflect the limited number of years driving the non‐linear relationship between survival and wind.

Extended foraging distances during the breeding season have previously been associated with lower rates of fecundity and declining population trends in a diving seabird, the Atlantic puffin (*Fratercula arctica*, Fayet et al., 2021). Population growth in long‐lived species, such as seabirds, is considerably more sensitive to changes in adult survival, compared to fecundity (Horswill et al., 2021; Sæther & Bakke, 2000; Stearns, 1992). A link between extended foraging trips during the breeding season and lower subsequent rates of adult survival, thus presents an additional mechanism that could mediate interpopulation differences in both seabird demography and population dynamics. In our study, we propose that individuals from colonies that conduct on average longer foraging trips experience greater reproductive costs and carry‐over effects leading to lower subsequent rates of adult survival, particularly during years with adverse conditions.

Despite identifying a small difference in mean rates of adult survival between Copeland and Skomer Island (1%), we show that the slightly higher and less variable time series of survival imputed for Manx shearwaters breeding on Copeland generates considerably faster rates of population growth. This finding agrees with previous studies demonstrating that small differences in seabird rates of adult survival can lead to quantitative population‐level effects (Horswill et al., 2021). A demographic difference of 1% is used by the EU ORNIS committee (EU Birds Directive 79/409/EEC) as a threshold for acceptable additional mortality that results in negligible population effects. However, this is not a consensus view (Horswill, Miller et al., 2022; Schippers et al., 2020), and, in agreement, we show that a difference of 1% in mean rates of adult survival can dramatically alter long‐term population trajectories.

We demonstrate that the imputed times series of adult survival can generate rapid population growth. This analysis does not reflect true population change on Copeland or Skomer Island. This is because we do not account for individual heterogeneity in survival, fecundity or breeding propensity, and juvenile survival is modelled as a function of adult survival. Furthermore, to standardise the analysis, we apply a constant rate of fecundity and breeding propensity to both colonies. Typically, fecundity is highly temporally (Horswill & Robinson, 2015) and spatially (Horswill et al., 2021) variable in seabirds. Environmental stochasticity can also increase the demographic impact of demographic rates that typically have a low influence on population growth, such as fecundity in long‐lived species (Gaillard et al., 1998). Incorporating temporal variation and directional trends in fecundity could therefore increase predictive accuracy in these analyses (e.g. Horswill, Miller et al., 2022).

Previous analysis based on a shorter time series than considered in our study (1993–2019, Wood et al., 2021), proposes that Manx shearwater survival declines with increasing summer wind speeds in the breeding season foraging area. By contrast, we identified that a negative relationship with summer wind speed only occurs above wind speeds of approximately 3.04 m/s. The detection of reproductive costs only during elevated environmental conditions is reported in terrestrial mammals and birds (Festa‐Bianchet & Jorgenson, 1998; Laaksonen et al., 2002; Mysterud, Stenseth, et al., 2001; Mysterud, Yoccoz, et al., 2001; Tavecchia et al., 2005). However, to date, studies showing this relationship in marine species are limited.

Previous studies examining the flight behaviour of foraging Manx shearwaters from nearby breeding colonies (Bardsey and Lundy) suggest that individuals can achieve flight at very low duty cycles in perpendicular crosswinds (Spivey et al., 2014) and high wind speeds (~8 m/s, Gibb et al., 2017). This suggests that if individuals choose a bearing that ensures flight at 90° to the wind, on both the outward and return leg of a foraging trip, then interpopulation energetic differences associated with maximum foraging distances during the breeding season could be negligible. Consequently, physiological comparisons of individually tracked birds, with demographic monitoring and more nuanced climate data, such as the number of days with head winds above a critical wind speed value (e.g., 3 m/s), may further clarify interpopulation differences in energy expenditure and reproductive effort.

The level of interpopulation synchrony in rates of adult survival reported in our study (ICC = 0.87) is at the top end of values previously published for other species of seabird. For example, the mean intra‐class correlation (ICC) between populations of Atlantic puffin with strong spatial overlap during the non‐breeding season range from 0.79 to 0.87 (Reiertsen et al., 2021). By contrast, populations of Atlantic puffin that are more spatially distinct have ICC scores between 0.32 and 0.67 (Grosbois et al., 2009; Reiertsen et al., 2021). In these other studies, annual variation in survival was linked to the North Atlantic Oscillation (NAO), as well as mean wind speeds and sea surface temperatures (Grosbois et al., 2009; Reiertsen et al., 2021). Large‐scale climate indices, such as NAO, are commonly identified as indirect and direct drivers of seabird survival. For example, studies on other seabird species that spend the non‐breeding period in the south‐west Atlantic Ocean, similar to Manx shearwaters, have previously documented relationships between survival and the El Niño/Southern Oscillation (ENSO) phenomenon, the Southern Annular Mode and the Southern Oscillation Index (SOI) (e.g., Horswill et al., 2014; Pardo et al., 2017). Previous studies of survival in Manx shearwaters did not identify an influential effect from winter sea surface temperature, winter wind speed or SOI (Wood et al., 2021). However, ENSO variability generates anomalies in sea‐surface temperature that are advected over several years to the South Atlantic (Meredith et al., 2008). Consequently, it may be useful to consider whether ENSO and SOI under summed physical and biological process lags can describe some of the residual variation in Manx shearwater rates of survival.

The maximum foraging distances exhibited by colonial seabirds during the breeding season can vary interannually. For example, during years with adverse prey or climatic conditions, individuals may forage farther from the colony and extend trip durations (Burke & Montevecchi, 2009; Horswill et al., 2017). Our study considers two colonies where interpopulation differences in maximum foraging distances are reported based on 3 years of data (Dean et al., 2015). These data were collected in concurrent years where colony‐specific habitat utilisation did not vary interannually (Dean et al., 2015). Therefore, if Manx shearwaters extend their foraging trip distances and durations during years with low prey availability or adverse climatic conditions, similar to other species of seabird (Burke & Montevecchi, 2009; Horswill et al., 2017), additional tracking and energy budget analysis (e.g. Dunn et al., 2022), alongside the existing mark‐recapture monitoring, may further elucidate our proposed interpopulation differences in carry‐over effects.

The imputed mean rates of adult survival for Manx shearwaters breeding on Copeland and Skomer Island agree with previously published values for this species. For example, studies conducted in the 1960s estimate rates of adult survival for Manx shearwaters at another nearby colony (Skokholm Island) to be 0.95 (SD = 0.02; Harris, 1966). Similarly, previous multi‐state analysis of Manx shearwater survival on Skomer Island estimates a highly similar mean value for successful breeders (0.94, 95% CI 0.92–0.95, Wood et al., 2021). However, Wood et al. (2021) also identify that populations of Manx shearwaters are comprised of individuals with different demographic profiles, whereby successful reproduction is associated with elevated survival and breeding propensity. That we identify highly synchronised rates of survival between Copeland and Skomer Island could imply that the proportion of birds with each demographic profile is similar across colonies, although future work could benefit from examining this further.

In this study, we identify interpopulation differences in rates of adult survival between two colonies of Manx shearwater that differ in population size and maximum foraging distances. Rates of survival were slightly lower in the larger colony that exhibits longer foraging trips during the breeding season. We hypothesise that carry‐over effects associated with colony‐specific foraging distances impact subsequent rates of adult survival. We also demonstrate that the interpopulation differences in rates of adult survival could lead to interpopulation differences in population dynamics. Additionally, we found that temporal fluctuations in rates of adult survival were highly synchronous between the study colonies, and that summer wind speed only impacts rates of survival at elevated values. Our study highlights that processes occurring during the breeding season can be important for understanding interpopulation differences in the demography and population dynamics of long‐lived species, such as seabirds.

## AUTHOR CONTRIBUTIONS

**C. Horswill:** Conceptualization (lead); formal analysis (lead); investigation (lead); methodology (lead); software (lead); visualization (lead); writing – original draft (lead); writing – review and editing (lead). **V. Warwick‐Evans:** Formal analysis (supporting); methodology (supporting); writing – review and editing (equal). **N. P. G. Esmonde:** Data curation (equal); writing – review and editing (equal). **N. Reid:** Data curation (equal); funding acquisition (equal); writing – review and editing (equal). **H. Kirk:** Data curation (supporting); formal analysis (supporting); writing – review and editing (equal). **K. R. Siddiqi‐Davies:** Data curation (supporting); formal analysis (supporting); writing – review and editing (supporting). **S. A. Josey:** Data curation (equal); writing – review and editing (equal). **M. J. Wood:** Data curation (equal); funding acquisition (equal); project administration (lead); writing – review and editing (equal).

## CONFLICT OF INTEREST STATEMENT

The authors declare that they have no conflict of interest.

## Supporting information


Appendix S1

## Data Availability

For non‐commercial and academic use, shearwater ringing data are available on request from the Copeland Bird Observatory (CBO) (www.thecbo.org.uk) and the University of Gloucestershire Research Repository (https://eprints.glos.ac.uk). We are unable to share these data publicly without restriction due to potential commercial interests from energy developments in the seas around both colonies. Data requests for commercial use will be considered. Code to replicate the analysis is available in the Supporting Information.
