# Supplementary material for: Interpopulation differences and temporal synchrony in rates of adult survival between two seabird colonies that differ in population size and distance to foraging grounds
Source: Ecol Evol. 2023 Oct 3;13(10):e10455. doi: 10.1002/ece3.10455 (PMC10547933; doi:10.1002/ece3.10455)
Supplement: Supplementary file 1 — Appendix S1 [file ECE3-13-e10455-s001.docx]

## **Interpopulation differences and temporal synchrony in rates of adult survival between two seabird colonies****that differ in population size and distance to foraging grounds**

C. Horswill, V. Warwick-Evans, N.P.G. Esmonde, N. Reid, H. Kirk,

K. R. Siddiqi-Davies, S. A. Josey and M. J. Wood

**Supplementary Information**

**Appendix A1.** Assessing model fit

**Table S1.** Summary table for prior distributions

**Table S2.** Estimates of adult survival for Manx shearwaters breeding on Copeland and Skomer Island.

**Figure S1.** Representative distributions showing temporal variation on the observed scale for the minimum value (i.e., 0.1) of the prior distributions assigning the standard deviation of the global ( $\sigma_{\gamma}$) and local ( $\sigma_{\varepsilon}$) random effect terms for adult survival.

**Figure S2.** Trace plots for Manx shearwater adult survival at Copeland.

**Figure S3.** Trace plots for Manx shearwater adult survival at Skomer Island.

**Figure S4.** Trace plots of Manx shearwater recapture for individuals observed during the previous year at Copeland.

**Figure S5.** Trace plots of Manx shearwater recapture for individuals observed during the previous year at Skomer Island.

**Figure S6.** Trace plots for survival intercept values.

**Figure S7.** Trace plots for recapture intercept values.

**Figure S8.** Trace plots for the standard deviation of the random effect terms.

**Figure S9.** Wind speed (m/s) was highly correlated between the two NCEP-NCAR CDAS-1 grid squares (Fig. 1A, main text) used for estimating wind speed across the key breeding season foraging area for Manx shearwaters

**Figure S10.** Prior and posterior distributions for the slope terms describing the linear relationship between wind speed and survival for Manx shearwaters.

**Appendix A1.** Assessing model fit

To examine the influence of incorporating the synchronous random effect term on estimated rates of

apparent survival, we also ran independent CJS models for each colony (i.e., identical model structure but without $\gamma_{t}$, Eqn. 1). The survival estimates imputed by the state-space synchrony model were highly correlated with those imputed with independent colony models (Spearman correlation coefficient: 0.91 for Copeland and 0.96 for Skomer Island, Fig. A1).


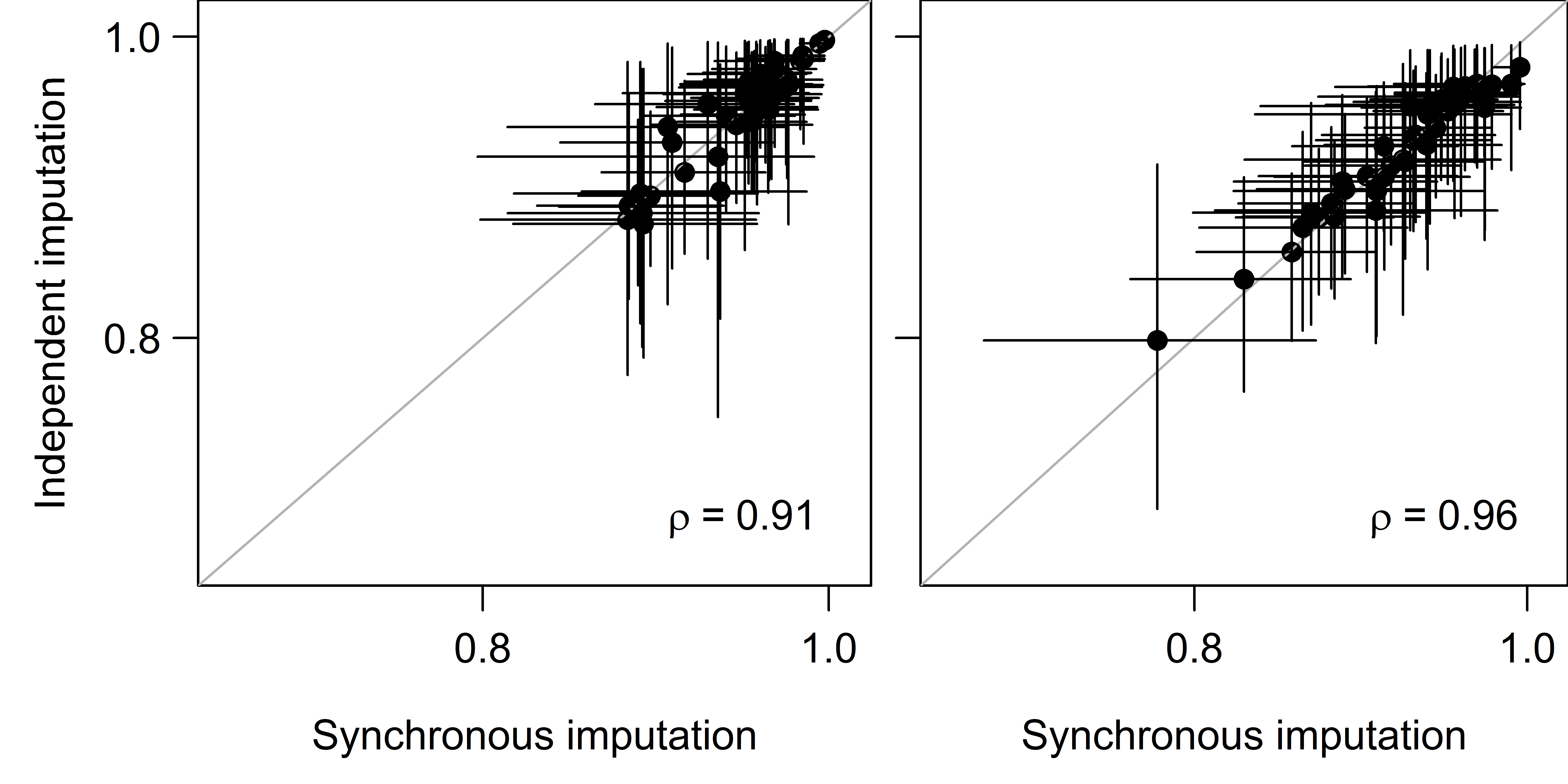


Fig. A1. The values of apparent adult survival imputed for each colony by the synchronous CJS model were highly correlated with the values estimated by the colony-specific fully time-dependent models. (A) Copeland (Spearman correlation coefficient = 0.91) and (B) Skomer Island (Spearman correlation coefficient = 0.96). Median posterior values shown with 95% CRI. Reference line shown at 1:1.

**Table S1.** Summary table for prior distributions used to impute rates of apparent survival and recapture of Manx shearwaters. The imputation scale for each parameter is indicated. Normal distributions detailed as $N(\mu,\sigma^{2})$

| Parameter | Notation | Prior | Scale |
| --- | --- | --- | --- |
| Intercept for adult survival | $a_{c}$ | $a'_{c}\sim U(0,1);a_{c}=\log\left(\frac{a'_{c}}{1-a'_{c}}\right)$ | $a'_{c}$Observed  $a_{c}$Logit |
| Synchronous temporal variation in adult survival |  | $N(0,\sigma_{\gamma}^{2})$ | Logit |
| Standard deviation of synchronous temporal variation in adult survival | $\sigma_{\gamma}$ | $\sigma_{\gamma}\sim U(0.1,10)$ | Logit |
| Asynchronous temporal variation in adult survival |  | $N(0,\sigma_{\varepsilon}^{2})$ | Logit |
| Standard deviation of asynchronous temporal variation in adult survival | $\sigma_{\varepsilon}$ | $\sigma_{\varepsilon}\sim U(0.1,10)$ | Logit |
| Intercept for recapture | $\delta_{k}$ | $\delta'_{c}\sim U(0,1);\delta_{c}=\log\left(\frac{\delta'_{c}}{1-\delta'_{c}}\right)$ | $\delta'_{k}$Observed  $\delta_{k}$Logit |
| Temporal variation in recapture | $\varepsilon_{p,c,t}$ | $N(0,\sigma_{p}^{2})$ | Logit |
| Standard deviation of temporal variation in in recapture | $\sigma_{p}$ | $\sigma_{p}\sim U(0.1,10)$ | Logit |

Notation: survival, $c$colony, $t$ time, $k$grouping for whether or not an individual was captured in the preceding year, $p$recapture.

**Table S2.** Estimates of adult survival for Manx shearwaters breeding on Copeland and Skomer Island. Median posterior value and 95% credible interval (CRI). Year represents survival from year $t-1$to year $t$; e.g., 1978 refers to survival between 1977 and 1978.

| Year | Copeland | | | Skomer Island | | |
| --- | --- | --- | --- | --- | --- | --- |
|  | 2.5% CRI | Median | 97.5% CRI | 2.5% CRI | Median | 97.5% CRI |
| 1978 | 0.99 | 1.00 | 1.00 | 0.98 | 1.00 | 1.00 |
| 1979 | 0.98 | 0.99 | 1.00 | 0.96 | 0.99 | 1.00 |
| 1980 | 0.93 | 0.97 | 0.99 | 0.81 | 0.91 | 0.98 |
| 1981 | 0.86 | 0.90 | 0.94 | 0.67 | 0.78 | 0.87 |
| 1982 | 0.95 | 0.98 | 1.00 | 0.90 | 0.97 | 1.00 |
| 1983 | 0.96 | 0.98 | 1.00 | 0.90 | 0.97 | 1.00 |
| 1984 | 0.90 | 0.95 | 0.99 | 0.84 | 0.94 | 0.99 |
| 1985 | 0.90 | 0.96 | 1.00 | 0.87 | 0.96 | 0.99 |
| 1986 | 0.90 | 0.96 | 0.99 | 0.88 | 0.96 | 0.99 |
| 1987 | 0.91 | 0.95 | 0.99 | 0.86 | 0.94 | 0.99 |
| 1988 | 0.92 | 0.97 | 0.99 | 0.88 | 0.96 | 0.99 |
| 1989 | 0.91 | 0.96 | 0.99 | 0.83 | 0.93 | 0.98 |
| 1990 | 0.91 | 0.95 | 0.99 | 0.82 | 0.91 | 0.97 |
| 1991 | 0.92 | 0.97 | 0.99 | 0.90 | 0.96 | 0.99 |
| 1992 | 0.93 | 0.98 | 1.00 | 0.92 | 0.97 | 0.99 |
| 1993 | 0.84 | 0.89 | 0.94 | 0.76 | 0.83 | 0.89 |
| 1994 | 0.92 | 0.96 | 0.99 | 0.85 | 0.91 | 0.97 |
| 1995 | 0.93 | 0.97 | 0.99 | 0.87 | 0.93 | 0.97 |
| 1996 | 0.91 | 0.96 | 0.99 | 0.87 | 0.93 | 0.98 |
| 1997 | 0.93 | 0.97 | 0.99 | 0.88 | 0.94 | 0.98 |
| 1998 | 0.89 | 0.94 | 0.98 | 0.84 | 0.90 | 0.96 |
| 1999 | 0.95 | 0.98 | 1.00 | 0.93 | 0.97 | 1.00 |
| 2000 | 0.93 | 0.98 | 1.00 | 0.92 | 0.97 | 0.99 |
| 2001 | 0.87 | 0.92 | 0.96 | 0.86 | 0.91 | 0.96 |
| 2002 | 0.93 | 0.96 | 0.99 | 0.91 | 0.95 | 0.99 |
| 2003 | 0.92 | 0.95 | 0.98 | 0.87 | 0.92 | 0.96 |
| 2004 | 0.91 | 0.96 | 0.99 | 0.90 | 0.95 | 0.98 |
| 2005 | 0.93 | 0.97 | 0.99 | 0.90 | 0.95 | 0.98 |
| 2006 | 0.83 | 0.88 | 0.94 | 0.82 | 0.87 | 0.92 |
| 2007 | 0.86 | 0.94 | 0.99 | 0.91 | 0.95 | 0.98 |
| 2008 | 0.82 | 0.89 | 0.96 | 0.84 | 0.89 | 0.94 |
| 2009 | 0.84 | 0.91 | 0.97 | 0.80 | 0.86 | 0.91 |
| 2010 | 0.86 | 0.93 | 0.98 | 0.83 | 0.88 | 0.94 |
| 2011 | 0.82 | 0.89 | 0.96 | 0.83 | 0.88 | 0.93 |
| 2012 | 0.88 | 0.95 | 0.99 | 0.88 | 0.93 | 0.98 |
| 2013 | 0.91 | 0.98 | 1.00 | 0.92 | 0.97 | 0.99 |
| 2014 | 0.81 | 0.89 | 0.96 | 0.82 | 0.89 | 0.95 |
| 2015 | 0.80 | 0.88 | 0.96 | 0.80 | 0.87 | 0.93 |
| 2016 | 0.81 | 0.91 | 0.97 | 0.80 | 0.87 | 0.94 |
| 2017 | 0.95 | 0.99 | 1.00 | 0.93 | 0.98 | 1.00 |
| 2018 | 0.80 | 0.94 | 0.99 | 0.84 | 0.93 | 0.99 |


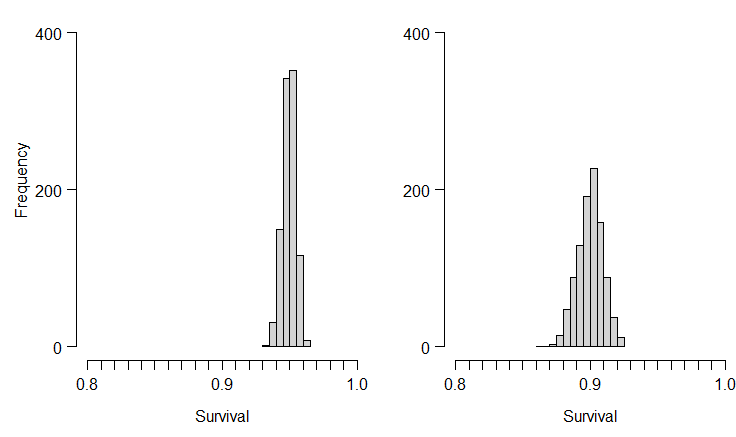
**Figure S1.** Representative distributions showing temporal variation on the observed scale for the minimum value (i.e., 0.1) of the prior distributions assigning the standard deviation of the global ($\sigma_{\gamma}$) and local ($\sigma_{\varepsilon}$) random effect terms for adult survival. A) Observed (back transformed) mean of 0.95 (logit value = 2.94) with an SD of 0.1 applied on the logit scale. B) Observed (back transformed) mean of 0.90 (logit value = 2.20) with an SD of 0.1 applied on the logit scale. In the CJS analysis, we assume that both the global and local random effect terms are independent of each other, and that total between year variance in survival equals $\sigma_{\gamma}+\sigma_{\varepsilon}$for each colony.


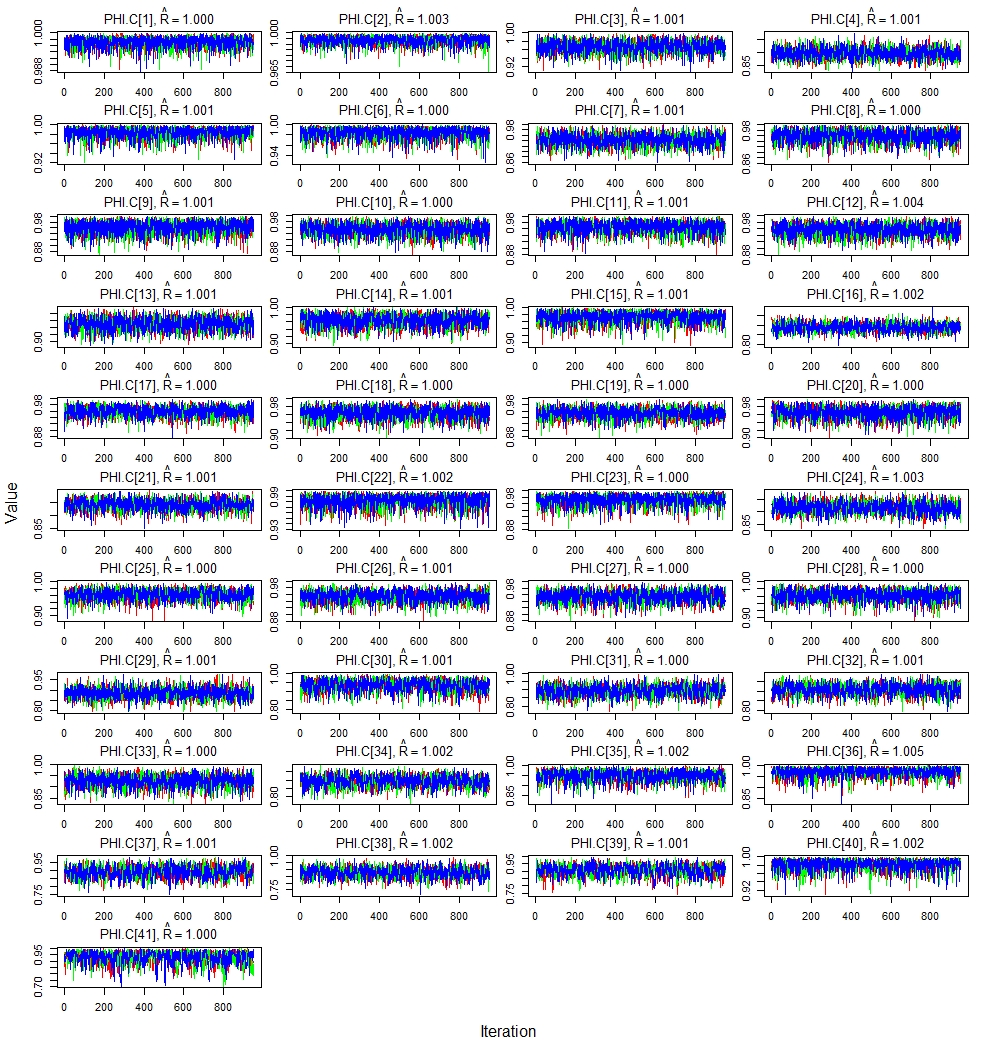


**Figure S2.** Trace plots for Manx shearwater adult survival at Copeland from the CJS model including wind speed. Plots included to demonstrate convergence of the MCMC chains.


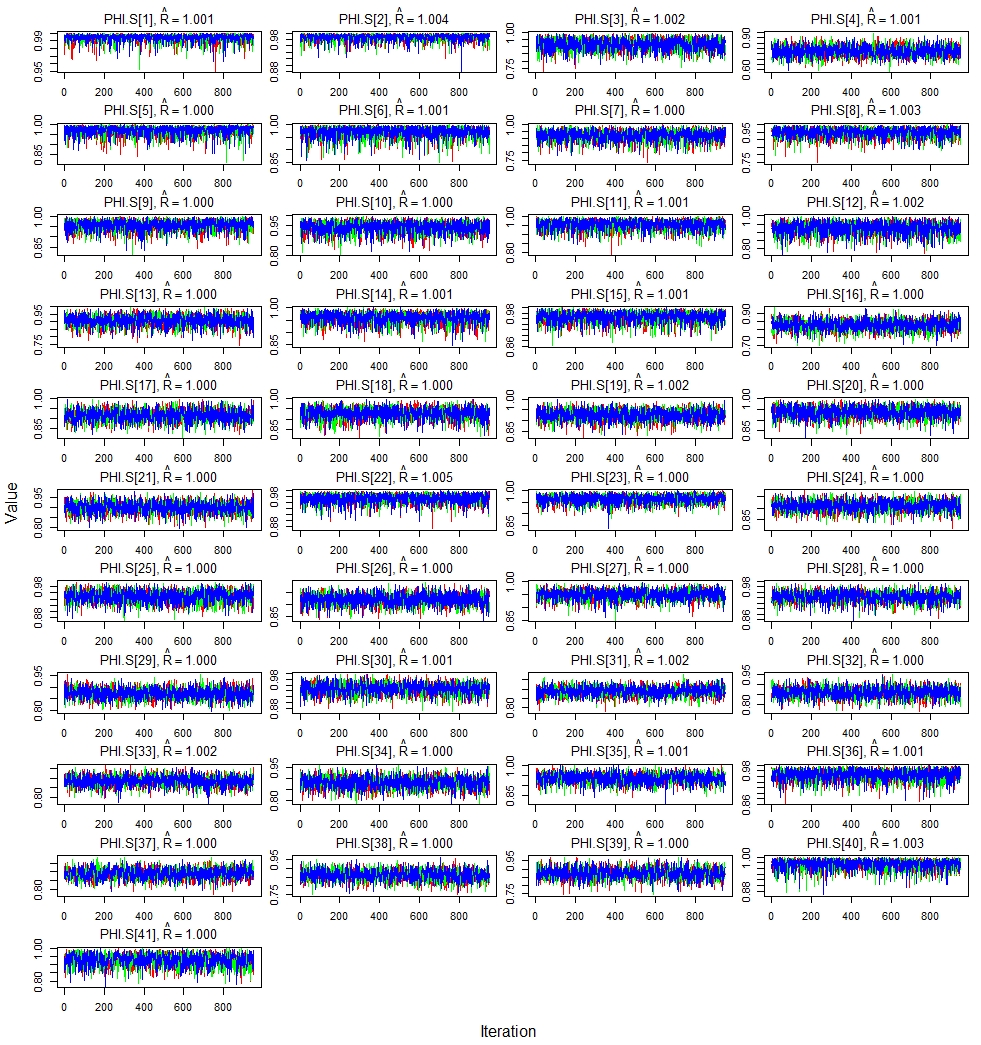


**Figure S3.** Trace plots for Manx shearwater adult survival at Skomer Island from the CJS model including wind speed. Plots included to demonstrate convergence of the MCMC chains.


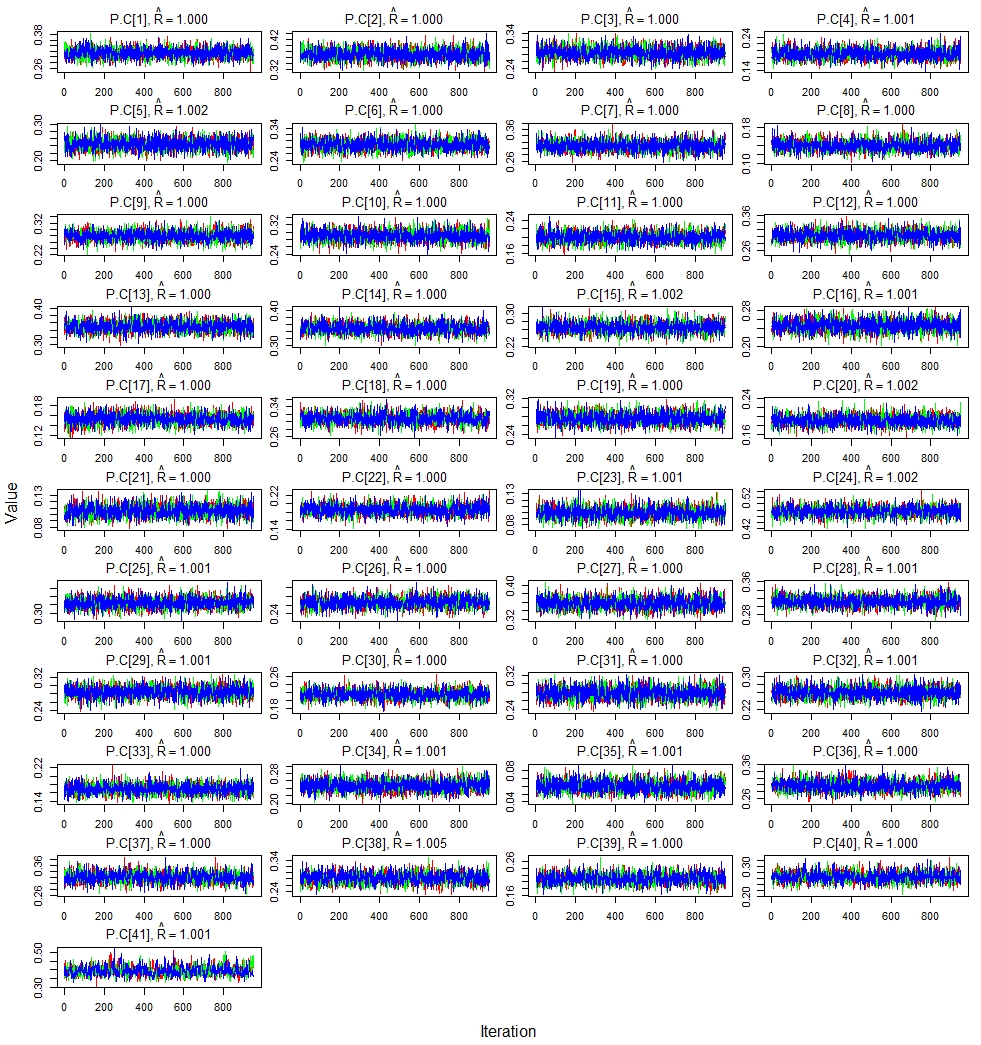


**Figure S4.** Trace plots of Manx shearwater recapture from the CJS model including wind speed for individuals observed during the previous year at Copeland. Plots included to demonstrate convergence of the MCMC chains.


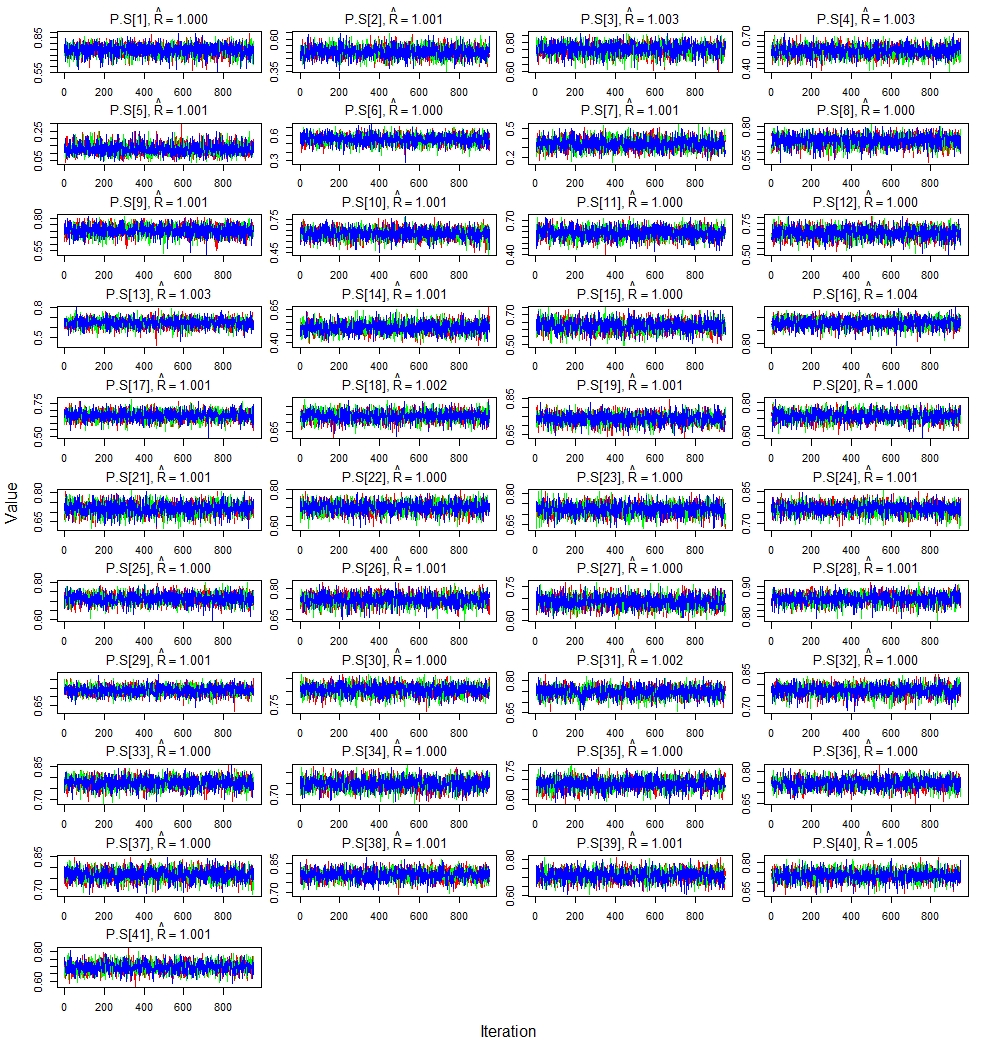


**Figure S5.** Trace plots of Manx shearwater recapture from the CJS model including wind speed for individuals observed during the previous year at Skomer Island. Plots included to demonstrate convergence of the MCMC chains.


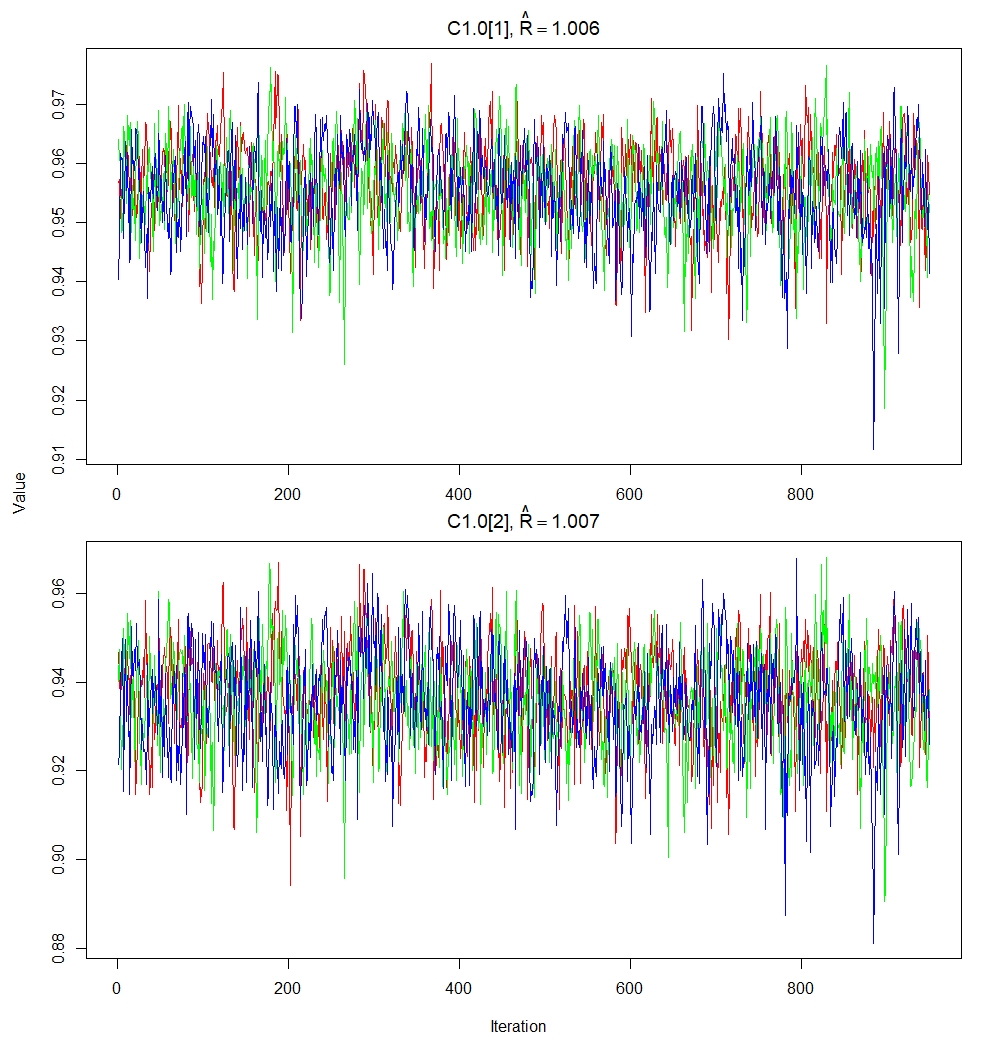


**Figure S6.** Trace plots for survival intercept values for the CJS model including wind speed. Top row: intercept value for adult survival at Copeland (C1[1]), intercept value for adult survival at Skomer Island (C1[2]). Plots included to demonstrate convergence of the MCMC chains.


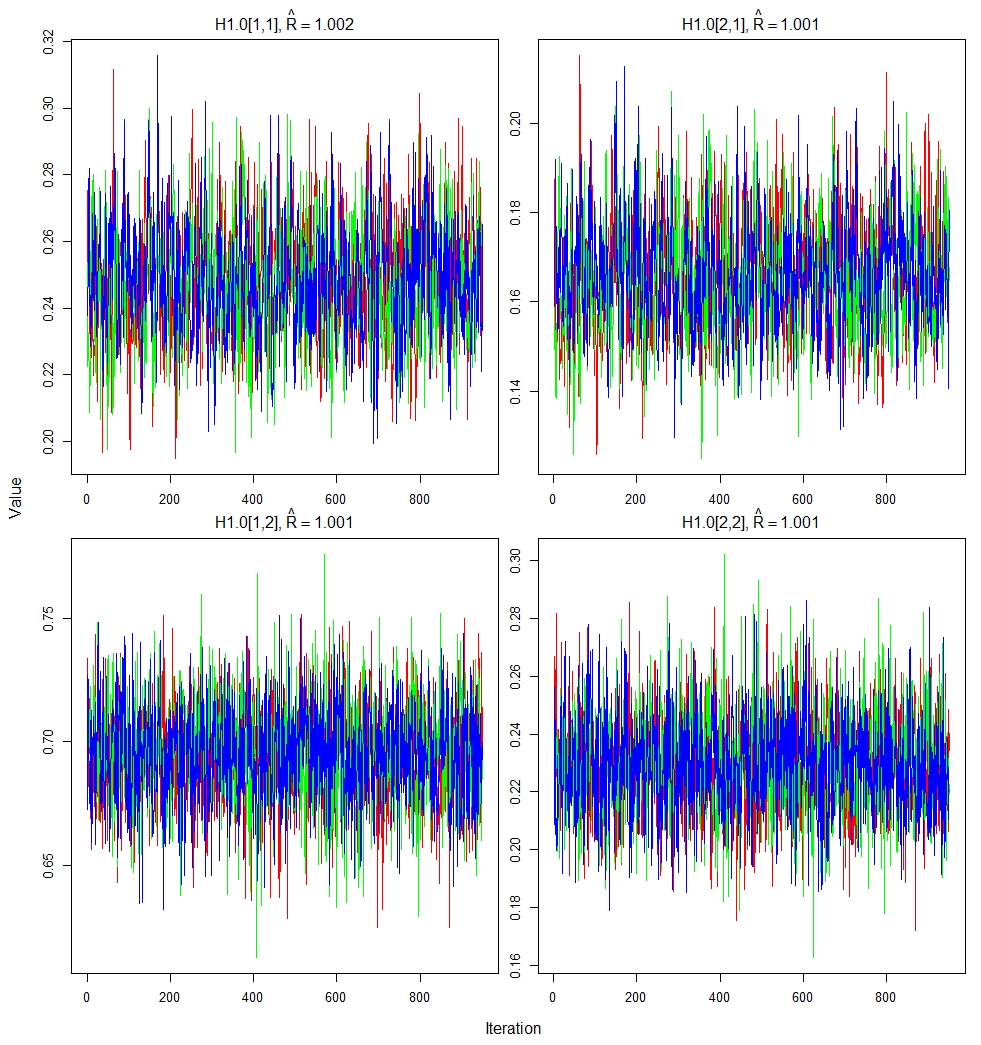


**Figure S7.** Trace plots for recapture intercept values for the CJS model including wind speed. Copeland if individual was not captured in the preceding year (H1[1,1]) and if individual was captured in the preceding H1[1,2], intercept values for recapture at Skomer Island if individual was not captured in the preceding year (H1[2,1]) and if individual was captured in the preceding (H1[2,2]). Plots included to demonstrate convergence of the MCMC chains.


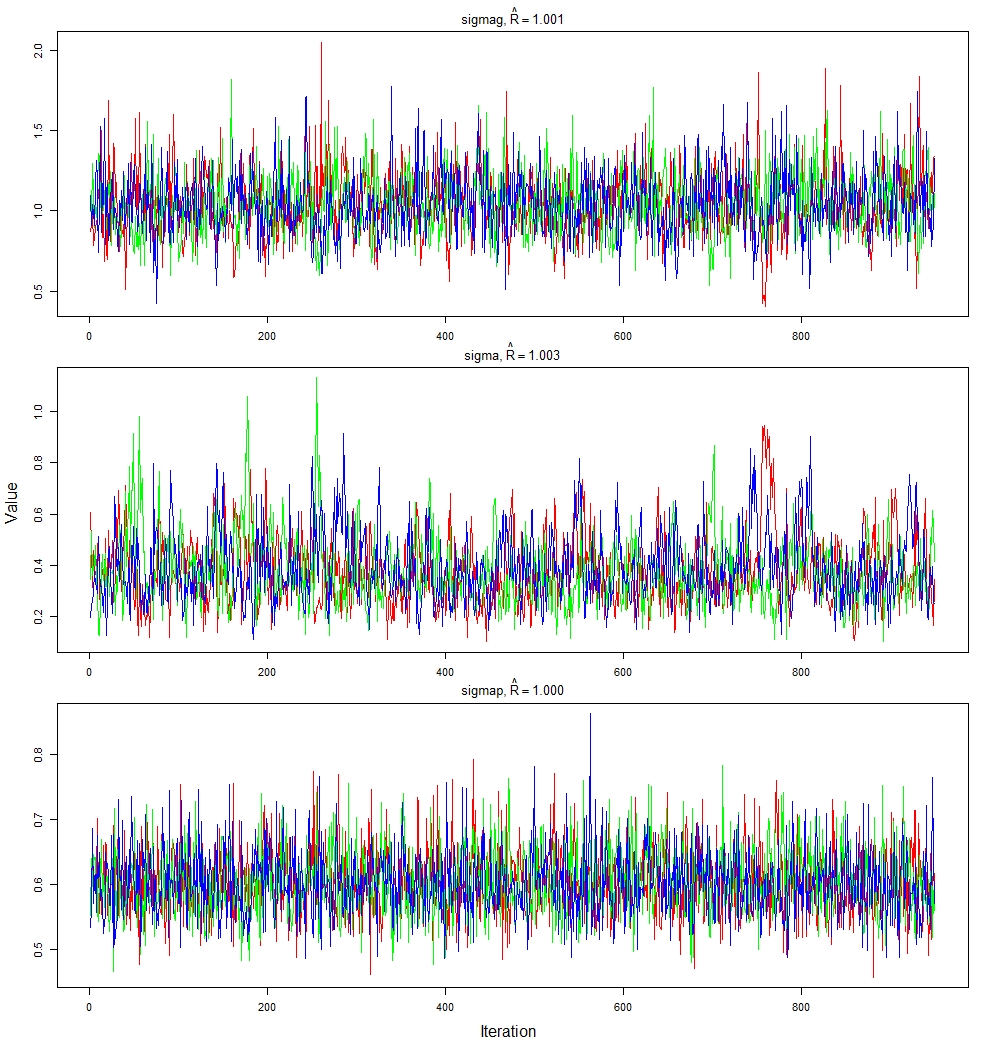


**Figure S8.** Trace plots for the standard deviation of the random effect terms for the CJS model including wind speed. Synchronous temporal variation in survival (sigmag), asynchronous temporal variation in survival (sigma) and temporal variation in recapture (sigmap). All trace plots shown on the logit scale. Plots included to demonstrate convergence of the MCMC chains.


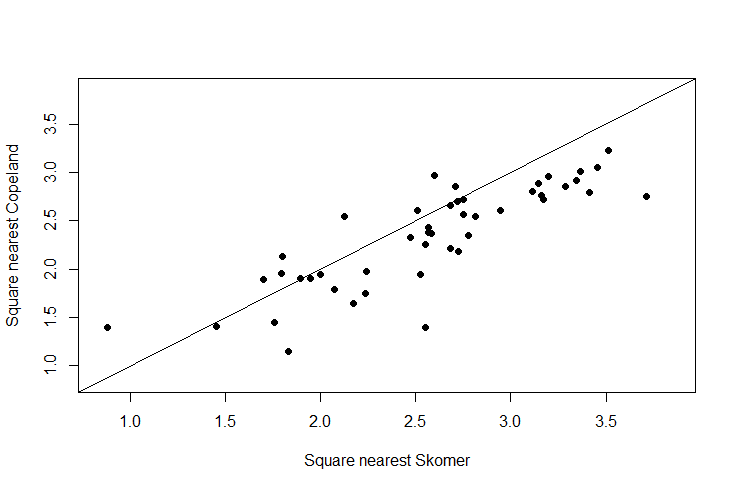


**Figure S9.** Wind speed (m/s) was highly correlated between the two NCEP-NCAR CDAS-1 grid squares (Fig. 1A, main text) used for estimating wind speed across the key breeding season foraging area for Manx shearwaters (Spearman correlation coefficient = 0.86). Wind speeds were slightly stronger in the square nearest Skomer Island.


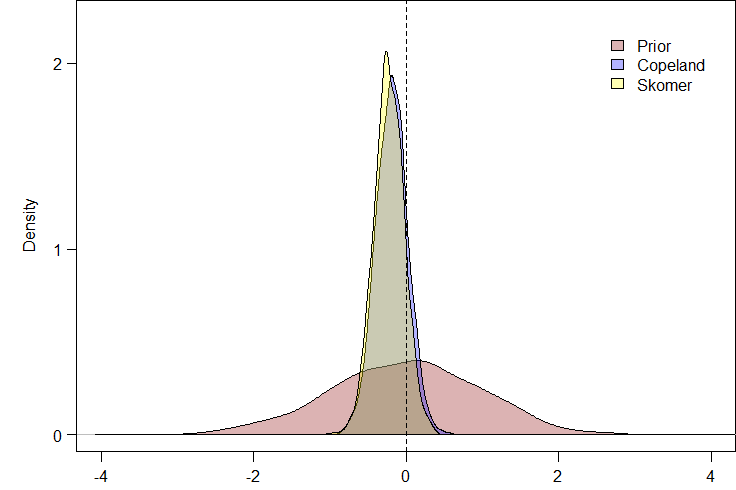


**Figure S10.** Prior (red) and posterior distributions for the slope terms describing the linear relationship between wind speed and survival for Manx shearwaters at Copeland (blue) and Skomer Island (yellow). Vertical dashed line at zero for reference.
